# pH-Responsive and Mucoadhesive Nanoparticles for Enhanced Oral Insulin Delivery: The Effect of Hyaluronic Acid with Different Molecular Weights

**DOI:** 10.3390/pharmaceutics15030820

**Published:** 2023-03-02

**Authors:** Shuangqing Wang, Saige Meng, Xinlei Zhou, Zhonggao Gao, Ming Guan Piao

**Affiliations:** 1 Key Laboratory of Natural Medicines of the Changbai Mountain, Ministry of Education, College of Pharmacy, Yanbian University, Yanji 133002, China; 2 State Key Laboratory of Bioactive Substance and Function of Natural Medicines, Department of Pharmaceutics, Institute of Materia Medica, Chinese Academy of Medical Sciences and Peking Union Medical College, Beijing 100050, China; 3 Department of Pharmacy, No. 73 Group Military Hospital of PLA, Xiamen 361003, China

**Keywords:** pH responsive, oral insulin delivery, hyaluronic acid, Caco-2 cell, pharmacodynamics

## Abstract

Drug degradation at low pH and rapid clearance from intestinal absorption sites are the main factors limiting the development of oral macromolecular delivery systems. Based on the pH responsiveness and mucosal adhesion of hyaluronic acid (HA) and poly[2-(dimethylamino)ethyl methacrylate] (PDM), we prepared three HA–PDM nano-delivery systems loaded with insulin (INS) using three different molecular weights (MW) of HA (L, M, H), respectively. The three types of nanoparticles (L/H/M-HA–PDM–INS) had uniform particle sizes and negatively charged surfaces. The optimal drug loadings of the L-HA–PDM–INS, M-HA–PDM–INS, H-HA–PDM–INS were 8.69 ± 0.94%, 9.11 ± 1.03%, and 10.61 ± 1.16% (*w*/*w*), respectively. The structural characteristics of HA–PDM–INS were determined using FT-IR, and the effect of the MW of HA on the properties of HA–PDM–INS was investigated. The release of INS from H-HA–PDM–INS was 22.01 ± 3.84% at pH 1.2 and 63.23 ± 4.10% at pH 7.4. The protective ability of HA–PDM–INS with different MW against INS was verified by circular dichroism spectroscopy and protease resistance experiments. H-HA–PDM–INS retained 45.67 ± 5.03% INS at pH 1.2 at 2 h. The biocompatibility of HA–PDM–INS, regardless of the MW of HA, was demonstrated using CCK-8 and live–dead cell staining. Compared with the INS solution, the transport efficiencies of L-HA–PDM–INS, M-HA–PDM–INS, and H-HA–PDM–INS increased 4.16, 3.81, and 3.10 times, respectively. In vivo pharmacodynamic and pharmacokinetic studies were performed in diabetic rats following oral administration. H-HA–PDM–INS exhibited an effective hypoglycemic effect over a long period, with relative bioavailability of 14.62%. In conclusion, these simple, environmentally friendly, pH-responsive, and mucoadhesive nanoparticles have the potential for industrial development. This study provides preliminary data support for oral INS delivery.

## 1. Introduction

Diabetes is a chronic metabolic disease that lasts a lifetime, especially type I diabetes, which requires frequent subcutaneous injections of insulin (INS) to maintain blood glucose balance [1,2]. However, such a pathway may lead to poor patient compliance and hypoglycemia. Oral INS delivery, as a more convenient treatment for diabetes, has the characteristics of simulating physiological INS secretion while overcoming these difficulties [3].

Whereas various biological barriers in the gastrointestinal tract hinder the clinical development of oral INS [4], oral INS delivery has to overcome these physiological barriers. First, orally delivered INS should avoid degradation by gastrointestinal enzymes and be stable over a wide pH range (gastric pH 1–3, enteral pH 6–7.5) [5]. Secondly, as a biomacromolecule, INS cannot directly enter the blood circulation through the intestinal mucosa, nor can it be straight internalized by intestinal epithelial cells into the blood [6]. Based on this, it is necessary to design a safe and effective drug carrier to deliver INS, which can overcome the harsh physiological environment of the gastrointestinal tract and be endocytosed into the blood circulation by intestinal epithelial cells.

With the continuous development of nanotechnology, there are many countermeasures to improve the bioavailability of oral INS [7,8]. pH-responsive delivery systems are the most widely reported INS carriers for targeted delivery and controlled release [9,10,11,12,13]. The pH-responsive delivery system protects INS from burst release and enzymatic degradation in gastric juices, and prolongs stability in vivo, thereby improving intestinal permeability and enhancing INS delivery efficiency [14].

pH-responsive polymers usually contain acidic or basic groups such as carboxyl, sulphonic acid, and amino groups. At different pH, pH-responsive polymers undergo protonation or ionization, changing the charge distribution and internal interactions, and thus changing the structure of the polymer [15]. Poly((2-dimethylamino)ethyl methacrylate) (PDM) is a well-known pH-sensitive polymer, and the presence of tertiary amine sites in its structure provides pH response characteristics [16]. The pKa of PDM is approximately 7.0. Thus, at pH < 7, PDM exhibits ionized forms of tertiary amine groups, and the polymer undergoes swelling, leading to a more efficient release of the loaded drug [17]. In this case, PDM acts as a cationic polymer. Thus, PDM can act as a carrier by interacting with anionic polymers, enzymes, or DNA through electrostatic interactions. Wang et al. [17] used photothermally sensitive polydopamine nanoparticles (NPs) modified with poly[(2-methacryloyloxy)ethyl phosphorylcholine-b-(2-dimethylamino)ethyl methacrylate] diblock copolymers to achieve near-infrared photothermal therapeutic and pH-sensitive drug release. Ghobashy et al. [18] prepared pH-responsive hydrogels using 2-(dimethylamino)ethyl methacrylate, polyethylene oxide, and ZnS NPs and investigated the release behavior of the hydrogels and the cumulative release of drugs increased from 40% at pH 4 to 96% at pH 7. Foss et al. [19] developed nanospheres of crosslinked networks of methacrylic acid grafted with poly(ethylene glycol), and acrylic acid grafted with poly(ethylene glycol) nanospheres for use as oral INS delivery devices. In addition, PDM contains carboxylic acid groups, which have the potential to open tight epithelial connections [20,21] and facilitate INS absorption. However, when PDM is used alone as a carrier to deliver drugs, it has a low drug loading capacity, unstable delivery system, and premature release, which may limit its further development [22,23,24]. The ideal drug delivery system should be versatile [14]. The introduction of another polymer is a feasible way to improve system stability and functional diversity [25].

The mucoadhesive delivery system adheres tightly to the epithelial mucosa of the intestinal, increasing the retention time of the preparation, and achieving the long-term stable release of drugs [26]. The rapid clearance of drugs at the gastrointestinal absorption site is also an oral absorption barrier [27]. Hyaluronic acid (HA) is a widely used adhesive carrier. HA, an anionic nonsulfated glycosaminoglycan natural polymer, is a viscous polysaccharide formed by the alternating connection of acetylglucosamine and glucuronic acid disaccharide [28]. HA is present in the extracellular matrix and synovial fluid of most human tissues and is also one of the main components of the extracellular matrix. It has many advantages as an adhesive carrier, such as ideal physicochemical properties, biocompatibility, non-immunogenicity, and biodegradability. The hydrophilic HA protects the activity of protein-based drugs and releases them slowly, making it a promising drug carrier. Using the negative charge carried by HA, it is self-assembled with cationic PDM using electrostatic interactions to form a new system for oral delivery of INS, which overcomes the disadvantages of rapid degradation and poor stability and improves the stability and bioavailability of INS in vivo.

The molecular weight (MW) of HA ranges from 10^3^ to 10^7^ Da, and different MW of HA has different properties [29]. The MW of HA should be chosen carefully because if polymers with higher MW are used, the release of peptides may be delayed due to high steric hindrance and high viscosity [30]. The accumulation of HA and the HA-binding proteoglycan versican around smooth muscle cells suggests that these molecules play an important role in cell proliferation and migration [31]. Segura et al. [32] prepared hydrogels using 1.33 × 10^6^ Da of HA with poly(ethylene glycol) diglycidyl ether using a crosslinking strategy. The hydrogel has low water content, a slow degradation rate, and ideal mechanical properties that could enhance its application in tissue engineering. Chiesa et al. [33] explored the role of the MW of HA (280, 540, 820 kDa) in the uptake of HA-based nanoparticles. In addition, some reports do not specify the MW of HA [34,35]. These result in low accuracy of experimental results and poor reference of the literature. Therefore, the effects of the MW of HA on carrier properties were explored in this study.

Due to the pH variation in the gastrointestinal tract, the pH responsiveness and mucosal adhesion of PDM and HA were combined to prepare the HA–PDM nano-delivery system loaded with INS. Firstly, self-assembled composite NPs, HA–PDM, were formed using the electrostatic interaction between the anionic polymer HA and the cationic polymer PDM. Then, the structural characteristics of NPs were determined using FT-IR. The preparation process of HA–PDM was optimized by a single-factor approach, and the effect of the MW of HA on the drug delivery system was investigated by particle size and zeta potential. After that, the release characteristics of INS in vitro were studied in 1.2, 6.8, and 7.4 pH PBS. The biocompatibility of NPs was also studied using CCK-8 and live–dead cell staining. Next, the ability of NPs to promote INS penetration was examined with Caco-2 monolayer cells and an ex vivo intestinal permeation experiment. Finally, in vivo pharmacodynamic and pharmacokinetic studies were performed to characterize hypoglycemic effects in diabetic rats following oral administration.

## 2. Materials and Methods

### 2.1. Materials

INS (I828365, derived from bovine pancreas, 27 u/mg), HA (1–2 × 10^5^, 4–8 × 10^5^, 1.5–2.5 × 10^6^), and potassium persulfate (P823296, 99.9%) were obtained from Shanghai Macklin Biochemical Technology Co., Ltd. (Shanghai, China). The measured MW of the three HA were 1.5 × 10^5^, 5 × 10^5^, and 2.2 × 10^6^, respectively. (2-dimethylamino) ethyl methacrylate (D111129, 99%), pepsin from porcine stomach (P128678, ≥2500 units/mg dry weight), and trypsin from bovine pancreas (T105531, potency ≥ 2500 units/mg) were purchased from Shanghai Aladdin Biochemical Technology Co., Ltd. (Shanghai, China). Insulin–FITC (MB5260, 7.14 mg/mL Insulin, 59.45 μg/mL FITC) was obtained from Dalian Bergolin Biotechnology Co., Ltd. (Dalian, China). Calcein AM Cell Viability Assay Kit (C203M, Lot No. 120121230207) was purchased from Shanghai Beyotime Biotech. Inc. (Shanghai, China). The other experimental reagents were of analytical grade and used without purification.

Human intestinal cell lines Caco-2 were obtained from the Cell Resource Center, IBMS, CAMS/PUMC and passages 30–40 were used. Caco-2 cells were cultured in 75 cm^2^ T-flasks in Dulbecco’s Modified Eagle Medium (DMEM) supplemented with 10% (*v*/*v*) fetal bovine serum (FBS), 1% (*v*/*v*) of a penicillin–streptomycin antibiotic blend, and 1% (*v*/*v*) glutamine in 37 °C, 5% CO_2_. Male Sprague–Dawley (SD) rats (160–180 g) were obtained from Vital River Laboratory Animal Technology Co., Ltd. (Beijing, China). All animal experiments were approved by the Laboratory Animal Ethics Committee in the Institute of Materia Medica and Peking Union Medical College. All procedures followed ethical standards during the experiment [36].

### 2.2. Preparation of NPs

The pH-responsive NPs were prepared by self-assembly of HA and 2-(Dimethylamino)ethyl methacrylate (DM). According to the single factor type in Table 1, the optimal prescription and the best process were screened. HA with three different MW was dissolved in distilled water, and some DM was added with stirring. The pH of the mixed solution was adjusted to 6.0 using hydrochloric acid. The initiator was added, and initiator was potassium persulfate (KPS) with a concentration of 2.5% (*w*/*v*) to initiate the polymerization of DM. Nitrogen was used as a protective agent. After a certain time at 65 °C, the reaction solution was removed and placed in a dialysis bag (8–10 KDa) and dialyzed for 72 h using distilled water to remove unreacted monomers. NPs were mixed with 2.5% (*w*/*v*) trehalose [37] and then frozen in a refrigerator at −40 °C overnight. Finally, the samples were lyophilized by using an EPSILON1-4LSC freeze dryer (Martin Christ, Osterode am Harz, Germany) to acquire freeze-dried NPs. Pressure (MPa) was 0.01 MPa. HA–PDM powder was obtained and stored in a moisture-proof cabinet. According to the different MW of HA, the three NPs were noted as L-HA–PDM, M-HA–PDM, and H-HA–PDM.

### 2.3. Physicochemical Properties of NPs

#### 2.3.1. FT-IR

The potassium bromide (KBr) pellet technique was used to obtain FT-IR spectra with Bruker Vertex 70 spectrometer (Bruker, Ettlingen, Germany). The samples were ground with KBr to prepare the pellets. The spectral width was 4000–400 cm^−1^, and the samples were scanned for 32 times.

#### 2.3.2. Particle Sizes and Zeta Potentials of NPs

Particle sizes, polydispersity indexes (PDI), and zeta potentials measurements were performed at 25 °C using a Zetasizer Nano ZS (Malvern, Worcestershire, UK) coupled with an MPT-2 accessory, fitted with a 532 nm laser at a fixed scattering angle of 173°. 1 mL of L-HA–PDM, M-HA–PDM, and H-HA–PDM (0.5 mg/mL) was filtered through a 0.8 μm syringe filter before performing the analysis [38]. The zeta potentials values (mV) were calculated from electrophoretic mobility using the Smoluchowski relationship (n = 3).

#### 2.3.3. Encapsulation Efficiency (EE) and Drug Loading (DL)

Firstly, the NPs loaded with INS were prepared. Briefly, a certain amount of INS hydrochloric acid solution (25, 50, 75, 100 mg) was first mixed with DM and then added dropwise to the HA solution. The optimal formulation and preparation process was used to obtain L-HA–PDM–INS, M-HA–PDM–INS, and H-HA–PDM–INS, respectively.

The DL and EE of the NPs were measured using an indirect method [39]. Briefly, after the INS-loaded NPs were prepared, the reaction solution was centrifuged at high-speed (18,000 rpm, 30 min, 4 °C). The reaction solution contained the INS-loaded NPs, unloaded INS (free INS), and unreacted monomers. At the end of the high-speed centrifugation, the upper layer of the solution contained unloaded INS and unreacted monomers. HPLC was used to determine the INS concentration in the upper solution. The specific operation of HPLC is documented in Supplementary Materials. The total amount of INS added minus the amount of free INS (unloaded INS) was the dose of NPs-loaded INS. DL and EE were calculated using Equations (1) and (2), respectively [40].

DL (%) = total amount of INS added − amount of free INS/total amount of INS added(1)

EE (%) = total amount of INS added − amount of free INS/weight of NPs(2)

#### 2.3.4. Transmission Electron Microscopy (TEM)

HT7700 Exalens Transmission Electron Microscopy (HITACHI, Chiyoda, Japan) was used to evaluate the roundness of NPs. The accelerating voltage was ×25.0 K Zoom-1 HC-1 80.0 Kv. The vacuum was less than 2 × 10^−5^ Pa. The NPs were uniformly dispersed in distilled water, and 6.0 μL sample was dropped onto a 300 mesh copper mesh and left for 5 min. Then, the excess liquid was blotted out with filter paper, dried, and observed, ×90,000.

#### 2.3.5. Storage Stability

The unloaded INS was removed using high-speed centrifugation (18,000 rpm, 30 min, 4 °C). After freeze-drying, the lyophilized NPs powder was obtained. Specific dialysis and freeze-drying process are described in Supplementary Materials.

The lyophilized NPs were stored at 4 °C and protected from light. The NPs were redissolved at different time points, and the particle size, PDI, and DL of the NPs were detected.

### 2.4. Stability of NPs in Simulated Gastrointestinal Fluids

The stability of L-HA–PDM–INS, M-HA–PDM–INS, and H-HA–PDM–INS was measured in simulated gastric fluid (SGF, pH 1.2) containing pepsin and simulated intestinal fluid (SIF, pH 7.4) containing trypsin. Proteins and peptides were highly susceptible to protease degradation in the gastrointestinal environment. SGF containing pepsin was prepared with 2.0 g sodium chloride and 3.2 g pepsin dissolved in water to 1000 mL, and pH was adjusted to 1.2 with hydrochloric acid. SIF containing trypsin was prepared with 6.8 g potassium dihydrogen phosphate, 77 mL 0.2 mol/L sodium hydroxide solution, and 10 g trypsin, then diluted to 1000 mL with water and adjusted pH to 7.4 with sodium hydroxide solution or hydrochloric acid solution.

It is well known that the preparation is in the stomach for 1–2 h and in the intestine for 2–4 h after oral administration. Briefly [41], 1 mL of SGF and SIF solutions were added to 20 mL of the test formulations (INS content equivalent to 1 mg) separately. The samples were incubated at 37 °C, 100 rpm. Free INS in the presence of SGF and SIF was incubated and used as a control. A hundred microliters of samples were collected at 0.25, 0.5, 0.75, 1, 1.5, and 2 h after adding SGF and 0.25, 0.5, 0.75, 1, 1.5, 2, and 4 h after adding SIF. Equal volumes were added to maintain the sink conditions. The degradation reactions of pepsin and trypsin were halted by the addition of 100 μL ice cold acetonitrile solution containing 0.1% (*v*/*v*) trifluoroacetic acid, respectively. The samples were extracted by incubation with pH 7.4 PBS and analyzed using HPLC to determine the amount of INS.

### 2.5. In Vitro Drug Release

#### 2.5.1. In Vitro INS Release

The in vitro INS release properties of NPs were determined by the dialysis diffusion technique under different pH conditions. The release medium included pH 1.2 SGF, pH 6.8 simulated colonic fluid, and pH 7.4 SIF. The NPs suspension was placed in a dialysis bag (MWCO 8–10 kDa) and then separately into the dissolution medium. At 37 °C, 50 rpm, the sample (1 mL) was taken out at the desired time points, and the fresh solution was added. INS concentration was measured using HPLC.

#### 2.5.2. Kinetics of Drug Release

The mechanism of INS release from NPs were determined using various kinetics models based on in vitro release data, which include zero-order, first-order, Ritger–Pappas, and Higuchi. The INS release percent (%) was plotted as a function of time as follows Equation (3):

(3) $$\frac{M_{t}}{M_{\infty }}=Kt^{n}$$

Here, *M_t_* was the amount of INS released in time *t*; *M*_∞_ was the total amount of INS released after unlimited time; *K* was the release rate constant; and *n* was the release index [42].

#### 2.5.3. Stability of INS in NPs

The secondary structure of INS released from NPs were determined by circular dichroism (CD) spectroscopy to examine the stability of INS in NPs. INS solution and INS released from NPs were prepared at pH 1.2, 0.05 M hydrochloric acid solution so that the final concentration of INS was 100 μg/mL. The scan range was 195–250 nm, the precision was 0.2 nm, the bandwidth was 0.5 nm, and the scan rate was 50 nm/min.

### 2.6. Mucin Adhesion and Ex Vivo Intestinal Permeation Study

#### 2.6.1. Mucin Adhesion

To evaluate the mucin adsorption and penetration of NPs, porcine mucin was mixed with an 8 mg/mL mucin reserve solution. The mixed solution was then mixed with NPs in equal volumes to obtain a 4 mg/mL NPs dispersion solution. After 1 h, the mixture was centrifuged at a high-speed of 13,000 rpm for 15 min, and the concentration of free INS in the supernatant was determined. The percentage of NPs with mucin aggregates was determined by calculating the ratio of free INS to total INS.

#### 2.6.2. Ex Vivo Intestinal Permeation Experiment

The absorption of NPs in vivo was simulated by an intestinal permeability experiment in vitro. The SD rats had fasted overnight. After inhalation of isoflurane at a concentration of 6% for 10 min, the rats were anesthetized and then sacrificed. From these rats, a total of 4 cm and a penetration area of 7.85 cm^2^ of the small intestine, with both ends ligated, was removed. The isolated intestines were used for ex vivo intestinal permeation experiment (quantitative experiment) and a confocal laser scanning microscope (qualitative experiment).

The 0.2 mL of 5 IU/mL INS solution, L-HA–PDM–INS, M-HA–PDM–INS, and H-HA–PDM–INS were injected into the isolated intestine, and then the intestine was placed in 5 mL pH 7.4 PBS at 37 °C and 50 rpm stirring speed [40]. The permeation experiment met the sink condition. At 0.5, 1, 2, 3, 4, 5, and 6 h, 0.5 mL of the solution was removed and supplemented with a fresh isothermal solution. The concentration of INS was determined (n = 6). The apparent permeability coefficient (Papp) reflects the ability of the drug to cross the intestinal, which is the drug transport rate.

Papp = dQ/dt × A × C_0_ (4)

Here, dQ/dt was the steady state rate of permeated INS over time. A was the diffusion area. C_0_ was the initial concentration of INS.

#### 2.6.3. Confocal Laser Scanning Microscope (CLSM)

Fluorescently labeled INS (INS–FITC) was used instead of INS. L-HA–PDM–INS–FITC, M-HA–PDM–INS–FITC, and H-HA–PDM–INS–FITC were prepared using the same method as for HA–PDM–INS. Briefly [43], the 0.2 mL of 5 IU/mL INS–FITC solution, L-HA–PDM–INS–FITC, M-HA–PDM–INS–FITC, and H-HA–PDM–INS–FITC were injected into the isolated intestine, and then the intestine was placed in 5 mL pH 7.4 PBS at 37 °C and 50 rpm stirring speed. At 1 and 2 h, the intestines were removed and rinsed separately. The intestines were dehydrated, embedded, and sectioned (4 μm). Finally, intestines were stained with 4′,6-diamidino-2-phenylindole (DAPI), and the penetration was observed with a Cytation5 (Biotek, Winooski, VT, USA) (n = 3). The excitation wavelength was 495 nm and the emission wavelength was 525 nm.

### 2.7. Caco-2 Cells

Caco-2 cells were used for assessment of the cytotoxicity and transintestinal epithelium delivery due to their morphological and functional similarity to the intestinal epithelium [44].

#### 2.7.1. CCK 8

CCK-8 assays were performed to evaluate the cytotoxicity of NPs on Caco-2 cells. In brief, Caco-2 cells were inoculated in 96-well plates for 24 h and 1 × 10^4^ cells/well. After the cells were attached to the walls, the medium was discarded and replaced with 100 μL of fresh DMEM containing different concentrations of NPs (50, 100, 200, 300, 400, 500 μg/mL), which were cultured in an incubator for 24 h. Then, the medium was discarded and replaced with serum-free DMEM containing 10% (*v*/*v*) CCK-8 solution. After 1.5 h of incubation, the OD value of every well was measured at a wavelength of 450 nm using a Synergy H1 microplate reader (Biotek, Winooski, VT, USA).

#### 2.7.2. Live–Dead Cell Staining

The survival of cells can be visualized using live–dead cell staining. The Caco-2 cell suspension concentration was adjusted to 1 × 10^5^ cells/well. Cells were seeded in 12-well plates and cultured for 1 d. Then, cells were treated with a DMEM medium containing different samples for 24 h. Live cells were stained with 1 μmol/mL calcein AM, and dead cells were stained with 1 μmol/mL propidium iodide. The images were taken using a CLSM.

#### 2.7.3. Transwell

Corning^®^ (Transwell pore diameter 0.4 μm, area 1.12 cm^2^) inserts with a 12-well plate were used to estimate the permeability. A quantity of 0.5 mL cell suspension was added to the donor chambers of the transwell plate at a density of 6 × 10^5^ cells/well, and 1.5 mL fresh DMEM containing serum was added to the receptor chambers. The transwell plates were cultured in a C170 CO_2_ incubator (Binder, Neckarsulm, Germany). The transepithelial electrical resistance (TEER) was measured each time the medium was replaced. TEER, which is formed by the flow of ions through the paracellular space, has become a common indicator for detecting the integrity of cell monolayers due to its simple operation and repeatable measurement.

Once the Caco-2 cell monolayer was fully established, the medium of the donor chambers and the receptor chambers was discarded. 0.5 mL NPs or INS solution was added to the donor chambers and 1.5 mL HBSS buffer was added to the receptor chambers, respectively. The transwell plates were placed in a CO_2_ incubator, and 200 μL solution was removed from the receptor chambers at different time points. At the same time, 200 μL HBSS was added.

#### 2.7.4. Cellular Uptake of NPs

CLSM can be used to observe the distribution of drugs in cells and qualitatively characterize cellular uptake. In brief, Caco-2 cells were seeded in 6-well plates at a density of 2 × 10^5^ cells/well. The well plates were incubated for 24 h in a CO_2_ incubator. INS–FITC-loaded NPs with a concentration of 50 μg/mL were prepared using free DMEM and divided into INS–FITC, L-HA–PDM–INS–FITC, M-HA–PDM–INS–FITC, and H-HA–PDM–INS–FITC. After 1 and 2 h of culture, the medium was removed, and the cells were washed three times with PBS. One milliliter of 4% paraformaldehyde solution was added to each well and fixed for 10 min. Finally, one milliliter of 1 μm/mL DAPI was added to each well and stained for 5 min. Cellular uptake was observed using a CLSM at 20×.

### 2.8. In Vivo Hypoglycemic Effect

#### 2.8.1. Establishment of the Diabetic Rat Model

Male SD rats were intraperitoneally injected with 60 mg/kg streptozocin (STZ, citrate-buffered saline, pH 4.5) to establish the diabetic model. One week later, the fasting blood glucose of SD rats was monitored. When the fasting blood glucose level of the model rats remained higher than 16.7 mmol/L for a week, it was considered that the diabetic rat model was successfully established and could be carried out in vivo experiments.

#### 2.8.2. Pharmacokinetics and Pharmacodynamics

SD rats were deprived of food for 6 h before the experiment. Thereafter, rats were randomly grouped (n = 6) and separately administered with L-HA–PDM–INS, M-HA–PDM–INS, and H-HA–PDM–INS by oral gavage (40 IU/kg), or subcutaneous (SC) injection (5 IU/kg). As a control group, another group of rats was set up with free INS (40 IU/kg) by oral gavage. Plasma was collected from the fundus venous plexus and the changes in blood glucose were analyzed using a glucometer (Haier, Qingdao, China).

INS concentrations in serum were measured using the Insulin Assay Kit (Nanjing Jiancheng, Nanjing, China). Relative bioavailability (F, %) was calculated according to Equation (5):

F% = AUC_(oral)_ × Dose_(S.C.)_/AUC_(S.C.)_ × Dose_(oral)_ × 100% (5)

Here, AUC_(oral)_ was the area under the drug–time curve for the oral group, AUC_(S.C.)_ was the area under the drug–time curve for the injection group, Dose_(oral)_ was the dose administered for the oral group, and Dose_(S.C.)_ was the dose administered for the injection group.

### 2.9. In Vivo Toxicity

The treatment of chronic diseases needs to be carried out over a long time. Pharmacological safety is a key factor to be considered for innovative preparations. To determine the in vivo safety of NPs, healthy SD rats were orally administered 60 IU/kg INS of NPs for 7 d [45]. The L-HA–PDM–INS, M-HA–PDM–INS, and H-HA–PDM–INS were administered according to the body weight of the rats on the day. After the last dose, plasma (1 mL) was collected from the fundus venous plexus using a capillary tube. The SD rats were sacrificed.

#### 2.9.1. Systemic Toxicity

The systemic toxicity of NPs were evaluated by detecting the serum levels of alanine aminotransferase (ALT), aspartate transaminase (AST), urea nitrogen (BUN), and γ-glutamyl transpeptidase (γ-GT) in rats. The serum was separated by centrifugation at 4 °C, 4000 rpm. Serum ALT, AST, BUN, and γ-GT were measured according to the instructions of the kit (Nanjing Jiancheng, China).

#### 2.9.2. Hemolysis Test

The blood compatibility of NPs were evaluated using spectrophotometry. Briefly, one milliliter of fresh rats’ blood was centrifuged at 4000 rpm for 10 min to separate red blood cells (RBCs). RBCs were washed three times with pH 7.4 PBS. The different concentrations of NPs were added to 2% (*v*/*v*) RBCs. Incubation was carried out at 37 °C for 1 h. Negative and positive controls were obtained by mixing RBCs with PBS and distilled water, respectively. Subsequently, the samples were centrifuged at 2000 rpm for 10 min. Approximately 100 µL supernatants for each sample were used to measure absorbance with a microplate reader (n = 3). Hemolysis was calculated using Equation (6).

Hemolysis (%) = (A_sc_ − A_nc_)/(A_pc_ − A_nc_) (6)

Here, A_sc_, A_nc_, and A_pc_ represent the absorbance of the sample, negative control (−) and positive control (+), respectively.

#### 2.9.3. Hematoxylin and Eosin Staining (H&E)

The heart, liver, spleen, lung, kidney, stomach, and small intestine of rats were fixed into 4% paraformaldehyde solution and stained for H&E. The safety of the NPs were evaluated using histopathology.

### 2.10. Statistical Analysis

All data were presented as mean ± standard deviation and experiments were performed at least three times. Statistical analysis was performed with the Prism 7.0 software (GraphPad Software) using Tukey’s multiple comparison tests and one-way analysis of variance (ANOVA). The differences were considered significant, when *p* values * <0.05, ** <0.01, and *** <0.001.

## 3. Results

### 3.1. Formulation Screening and Process Optimization

In order to obtain safe, effective, stable, convenient, and economical preparations, formulation screening and process optimization are often needed [46]. Table 2 showed the results of particle size, zeta potential, and PDI during formulation screening and process optimization. Different MW of HA (L, M, and H) were used to explore the effects of MW on the physicochemical properties of NPs. Regardless of the MW of HA, the particle size of NPs increased with increasing DM addition in the formulation screening range. The particle size of NPs were negatively correlated with the addition of KPS. Within the scope of process optimization, there was no obvious rule for the change of the particle size of NPs with different speeds, possibly because the speed at this level had no significant effect on the particle size. Regarding the reaction temperature, the reaction temperature was proportional to the MW of HA. However, the particle size of the obtained NPs were inversely proportional, and the change was obvious. The best formulation and process results were showed in Table 3. Therefore, the MW of HA has a slight influence on the preparation process of HA–PDM. When the MW of HA was larger, the dose of KPS and reaction temperature were higher. This may be because the viscosity of HA increases with the increase of MW, and the high dose of KPS and reaction temperature can slightly reduce the viscosity of the polymer.

### 3.2. Characterization of NPs

#### 3.2.1. FT-IR

**Figure 1:**
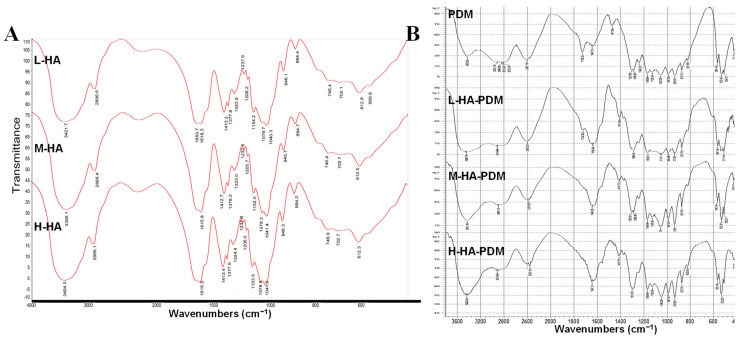
FT-IR. (**A**) HA. (**B**) PDM and HA–PDM.

Figure 1 showed the FT-IR results for the polymers and NPs. The stretching vibration absorption peak of the carboxyl group in HA appeared at 1616.35 and 1409.96 cm^−1^ [47]. The stretching peak near 2922.16 cm^−1^ belonged to -CH. The strong peak of 3385.07 cm^−1^ proved the existence of hydroxyl absorption (Figure 1A). The FT-IR of PDM showed two characteristic peaks at 1647.21 and 1728.22 cm^−1^, which were attributed to –C=C and –C=O, respectively, and 1350 cm^−1^ was the –CO stretch [18]. The peak located at 2848.86 cm^−1^ was attributed to the –CH bond stretching vibration of –N(CH_3_)_2_. In addition, the peak located at 2922 cm^−1^ was attributed to –CH stretching vibration (Figure 1B).

In the HA–PDM system, the absorption peaks at 1728.22 and 1647.21 cm^−1^ moved to 1724.36 and 1639.49 cm^−1^, respectively, and the absorption peaks were enhanced (Figure 1B). This was due to the existence of –C=C and –C=O [48]. In contrast, this peak was not evident in HA and PDM, indicating an electrostatic interaction between HA and PDM. At about 3400 cm^−1^, the peak shape was wide and blunt, showing intramolecular or intermolecular hydrogen bond association of hydroxyl groups, indicating that HA and PDM form NPs through a hydrogen bond, and the structure was relatively stable. This hydrogen bonding ability increased with increasing MW of HA.

#### 3.2.2. Morphology, Particle Sizes, and Zeta Potentials

**Figure 2:**
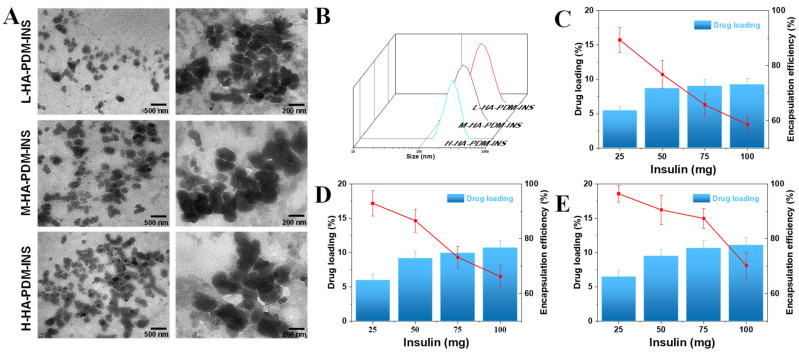
Characterizing data on the NPs. (**A**) TEM images of NPs, scale bar = 500 or 200 nm. (**B**) Dynamic light scattering of HA–PDM–INS. Drug loading and encapsulation efficiency of L-HA–PDM–INS (**C**), M-HA–PDM–INS (**D**), and H-HA–PDM–INS (**E**) (X ± SD, n = 3).

The TEM results (Figure 2A) showed that the three NPs had a nearly spherical structure with a compact structure and moderate dispersion. NPs were not completely monodispersed probably because the pH-sensitive NPs aggregate at pH 7.4 and above [49]. Some fragments were found in the TEM results (Figure 2A), which indicated that the pH-sensitive NPs also undergo swelling, relaxation, and erosion in neutral solutions for long time.

By electrostatic adsorption, HA and PDM self-assemble to form NPs. NPs with appropriate particle size are more likely to break through the spatial barrier of mucosa and promote further internalization of NPs into intestinal epithelial cells. After the addition of INS, the particle sizes of the three NPs increased (Figure 2B) because the addition of INS expanded the internal space of the HA–PDM system. The surface charge of NPs is a significant factor in determining the efficiency of cellular uptake, which affects the adhesion of NPs and interactions with organelles [50]. The three kinds of NPs prepared in this study all have high negative potentials. The presence of anionic polymers confers better adhesion to NPs because of the presence of many surface carboxyl groups on anionic polymers, which produce strong bioadhesive interactions with enteric epithelium through hydrogen bonding [51], improving the retention and penetration of NPs.

### 3.3. EE and DL

The EE and DL are characteristics of drug delivery systems and are vital for assessing the usability of the carrier. During agitation, HA–PDM adsorbs INS through hydrogen bonds or hydrophobic interactions. The DL and EE of NPs were indirectly determined using high-speed centrifugation (Figure 2C–E). When the total amount of INS was 25 mg, the DL of the L-HA–PDM–INS, M-HA–PDM–INS, and H-HA–PDM–INS was 5.41 ± 0.68%, 5.96 ± 0.85%, and 6.45 ± 0.93% (*w*/*w*), respectively. When the total amount of INS was 100 mg, the DL of the L-HA–PDM–INS, M-HA–PDM–INS, and H-HA–PDM–INS was 9.21 ± 0.95%, 10.65 ± 1.09%, and 10.05 ± 1.15% (*w*/*w*), respectively. When the total amount of INS was constant, the DL of HA–PDM–INS increased with an increase in the MW of HA. This is probably because HA with high MW occupies a large space and carries more carboxyl groups and negative charges [52]. Through charge interactions and hydrogen bonding, HA attracts a large amount of INS, increasing DL. Another phenomenon was also noted: the DL of the NPs increased with an increase in the INS dose, but the EE decreased significantly. Similar results have been reported previously [53,54]. The excessive INS perhaps approached the upper limit of the EE of the NPs and damaged the equilibrium of the loading process. When its loading capacity is exceeded, the INS can no longer be bound. Unloaded INS was dispersed in the free form in the reactive solution. The optimal amount of INS was determined by assessing the economic benefits. For the L-HA–PDM–INS and M-HA–PDM–INS, when the dosage was 50 mg, DL was 8.69 ± 0.94% and 9.11 ± 1.03% (*w*/*w*), EE was 76.81 ± 5.06% and 86.61 ± 4.21% (*w*/*w*), respectively. For the H-HA–PDM–INS, when the dosage was 75 mg, DL and EE were 10.61 ± 1.16% and 87.42 ± 3.66% (*w*/*w*), respectively.

### 3.4. Stability

**Figure 3:**
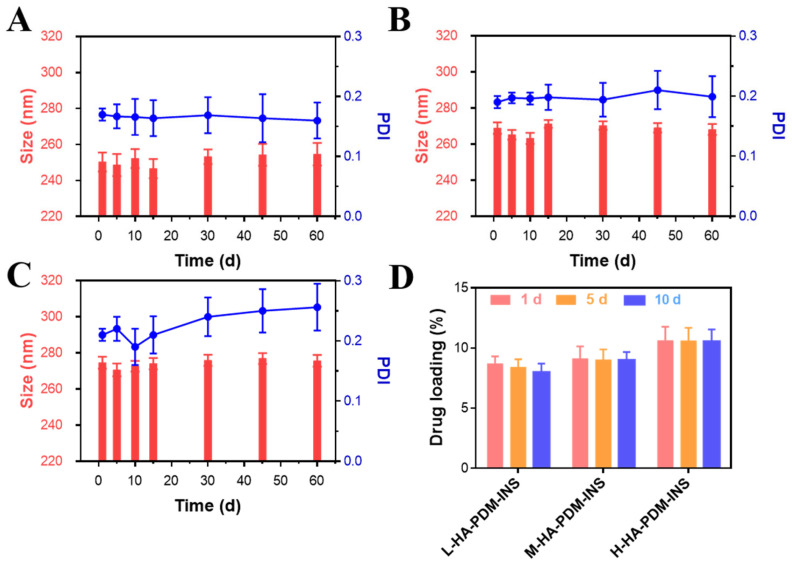
The size stability of L-HA–PDM–INS (**A**), M-HA–PDM–INS (**B**), and H-HA–PDM–INS (**C**) stored at 4 °C for 60 d. (**D**) Drug loading of three nanoparticles during storage (X ± SD, n = 3).

The stability of NPs plays a vital role. The M-HA–PDM–INS and H-HA–PDM–INS NPs prepared were stored at 4 °C for 60 d, and there was no significant change in particle size and INS leakage (Figure 3B,C). Nevertheless, there was a slight change in the L-HA–PDM–INS particle size (Figure 3A) and the DL decreased mildly over 60 d (Figure 3D). This indicated that L-HA–PDM–INS was slightly less stable compared to the NPs prepared from high MW of HA.

### 3.5. Anti-Protease Degradation Ability

**Figure 4:**
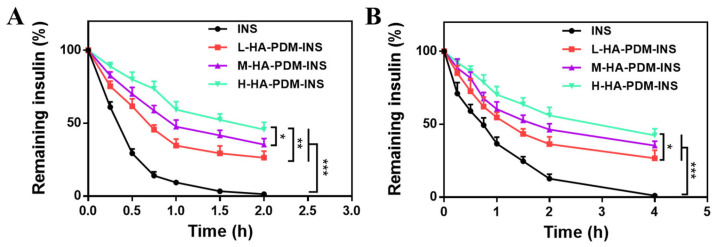
Stability profiles of the associated insulin after incubation of simulated gastric fluid containing pepsin (pH 1.2) (**A**) and simulated intestinal fluid containing trypsin (pH 7.4) (**B**) at 37 °C for various time points (X ± SD, n = 3, * *p* < 0.05, ** *p* < 0.01, *** *p* < 0.001).

A major problem in oral INS delivery is that INS is susceptible to degradation by proteases in the gastrointestinal tract, particularly trypsin and pepsin. The results also showed that free INS was rapidly and completely degraded in SGF containing pepsin and SIF containing trypsin (Figure 4). All three NPs significantly delayed the degradation of INS by protease. In particular, H-HA–PDM–INS showed the most significant resistance to protease degradation. The INS of H-HA–PDM–INS remained at approximately 50% retention at 2 h of pepsin action or 4 h of trypsin action, because the high MW of HA had a large viscosity, and the H-HA–PDM system was more stable, which tightly bound INS inside the NPs. The FT-IR results also showed that the H-HA–PDM system had higher hydrogen bonding (Figure 1B). The results of the antiprotease degradation experiments showed that after INS was encapsulated into the NPs, there was a certain “steric hindrance” between the NPs and the protease, which prevented the protease from contacting the INS and thus resisted the degradation.

### 3.6. In Vitro Release

#### 3.6.1. pH-Responsive Release of NPs

**Figure 5:**
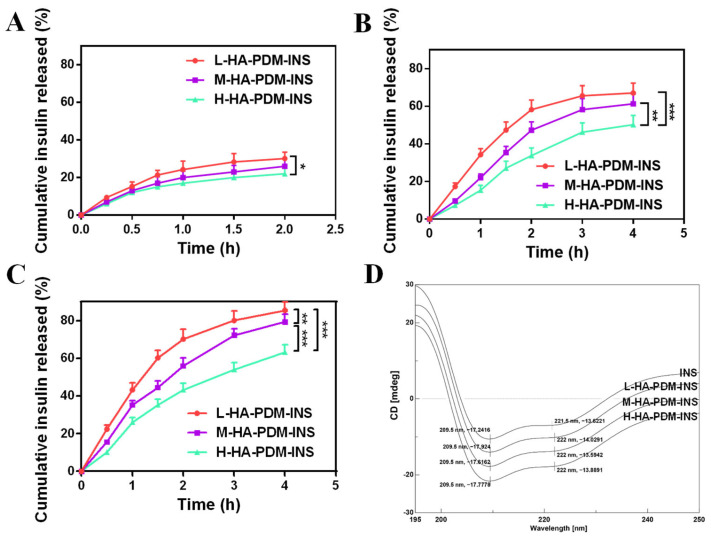
In vitro release profile of insulin from HA–PDM–INS in pH 1.2 (**A**), pH 6.8 (**B**), and pH 7.4 (**C**) PBS (X ± SD, n = 6, * *p* < 0.05, ** *p* < 0.01, *** *p* < 0.001). (**D**) Circular dichroism spectra of insulin released from three nanoparticles.

The intestine is the most critical site for the absorption of NPs. Oral preparations need to pass through the stomach to reach the intestine. The ability of the NPs to remain stable over a wide pH range plays an important role in the subsequent uptake of NPs by intestinal epithelial cells. Therefore, we investigated the INS release behavior of NPs in pH 1.2, 6.8, and 7.4 PBS, and the results are shown in Figure 5. At pH 1.2, all three NPs released only a small amount of INS within 2 h. The amount released was less than 30%, indicating that the NPs were more stable at pH 1.2 (Figure 5A). The cumulative release increases gradually with increasing pH (Figure 5B,C). Interestingly, at all three pH, the release of NPs were slower, and the cumulative release was lower as the MW of HA increased. The results are consistent with the results of NPs resistance to protease degradation. This is probably due to the high viscosity of the high MW of HA and the tighter internal network of NPs, which hindered the release of INS.

A modeling study of release was vital for appraising and predicting INS release behaviors in vitro and in vivo [55]. The R^2^ was used as an index of the best fit of NPs release model. The diffusional exponent depended on the release mechanism of the NPs delivery. In vitro release behaviors were fitted, as shown in Table 4. The release of INS was a non-Fickian diffusion (0.45 < n < 0.89) and erosion release mechanism. The swelling and relaxation of polymer chains, the ionization reaction between carrier and INS, and the invasion of the matrix polymer, were involved during the release process. This greatly increased pore size and allowed for the continuous release of INS.

#### 3.6.2. Structural Stability of INS

INS has an inherent α-helix conformation, which is essential for the interaction between INS and its receptor and the biological activity of INS. The α-helix and β-sheet of INS correspond to the presence of two negative characteristic peaks at 209.5 nm and 221.5 nm, respectively (Figure 5D). INS released from all three NPs also showed obvious negative peaks at 209.5 nm and 222 nm, indicating that spatial conformation did not change significantly and had complete physiological activity. This indicates that INS can maintain a complete secondary structure in the NPs and had structural stability. The slight change in the absorption peaks at 209 nm and 222 nm may be due to a change in the pH of the system during the preparation of the NPs, which affected the hydrogen bonding between the carbonyl and amino groups on the polypeptide chain in the α-helical structure and affected the stability of the hydrogen bonds.

### 3.7. In Vitro Intestinal Penetration Study

**Figure 6:**
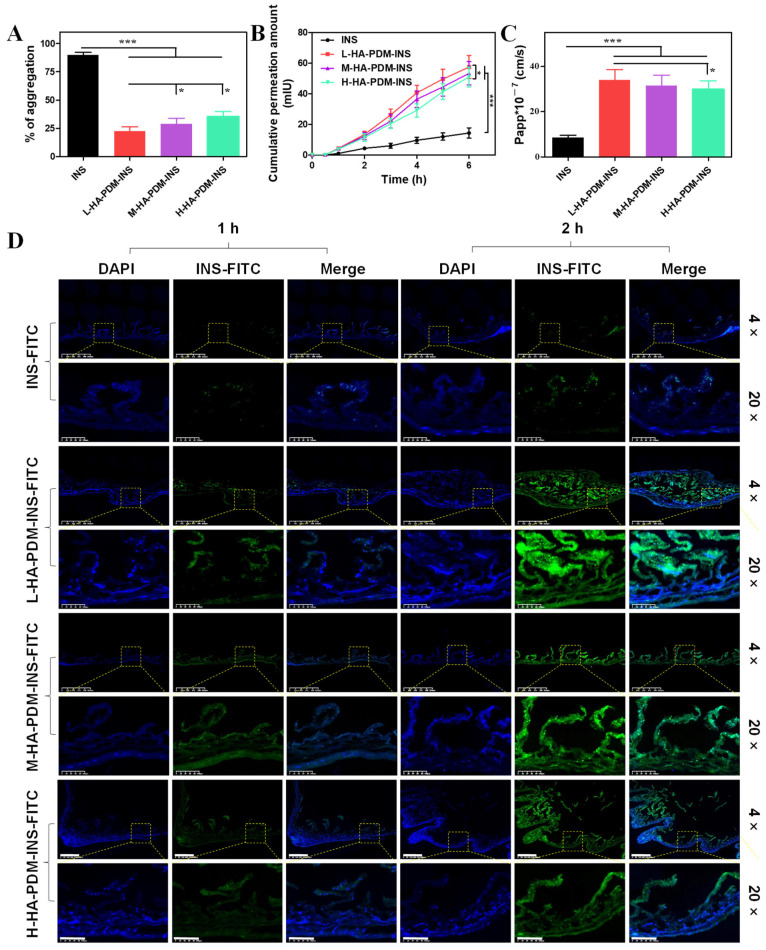
(**A**) Aggregates of free insulin, L-HA–PDM–INS, M-HA–PDM–INS, H-HA–PDM–INS, and mucins (X ± SD, n = 6, * *p* < 0.05, *** *p* < 0.001). (**B**) Permeation studies of insulin from L-HA–PDM–INS, M-HA–PDM–INS, and H-HA–PDM–INS across the intestinal loops (X ± SD, n = 6, * *p* < 0.05, *** *p* < 0.001). (**C**) Apparent permeability coefficient (Papp) of insulin across isolated intestinal sections (X ± SD, n = 6, * *p* < 0.05, *** *p* < 0.001). (**D**) Confocal micrographs of the small intestinal villi sections after incubated with different formulations for 1 and 2 h, scale = 100 μm.

The mucoadhesive and penetration of the oral NPs is a key feature that must be sequential and balanced with each other to avoid mucin trapping, with the aim of better uptake by enterocytes. The aggregation results of different NPs and mucins are shown in Figure 6A. The results showed that the aggregation of free INS reached 89.67 ± 2.52%, which was due to van der Waals forces, hydrogen bonds, and hydrophobic interaction between INS and mucin [50]. In contrast, the presence of the strongly negatively charged HA–PDM–INS hindered the adsorption of mucin, and the aggregations were reduced to 22.33 ± 4.04%, 29.00 ± 5.00%, and 35.33 ± 4.51%, respectively. The aggregation of L-HA–PDM–INS was significantly lower than that of M-HA–PDM–INS and H-HA–PDM–INS. L-HA–PDM–INS showed the best antimucin adhesion.

As the intestinal epithelium has been considered the most challenging barrier to the oral delivery of NPs, the present study further examined the ability of three NPs to permeate through the intestine in vitro. The cumulative permeation amount of the different NPs were time-dependent (Figure 6B). Among them, the cumulative permeability of L-HA–PDM–INS in the intestine was the highest, and the Papp was 34.01 ± 4.51 × 10^−7^ cm/s, which was significantly higher than that of H-HA–PDM–INS (Papp = 29.84 ± 3.85 × 10^−7^ cm/s) (Figure 6C).

The penetration of NPs loaded with INS–FITC can be visually seen using CLSM. The greater fluorescence intensity indicated a higher INS–FITC concentration. As shown in Figure 6D, L-HA–PDM–INS–FITC had the largest penetration depth at 2 h, which was consistent with the results of in vitro intestinal permeation studies.

### 3.8. Caco-2 Study

The Caco-2 cells aggregated in the logarithmic growth phase and continued to grow to differentiate into monolayer cells, forming a tight junction structure similar to the small intestine, which was consistent with the cell model for the study of oral drug absorption in vitro.

#### 3.8.1. Biocompatibility

For practical application of drug delivery systems, they must be nontoxic and have good biocompatibility. In this project, CCK-8 and live–dead cell staining were used to determine the biocompatibility of NPs in vitro.

**Figure 7:**
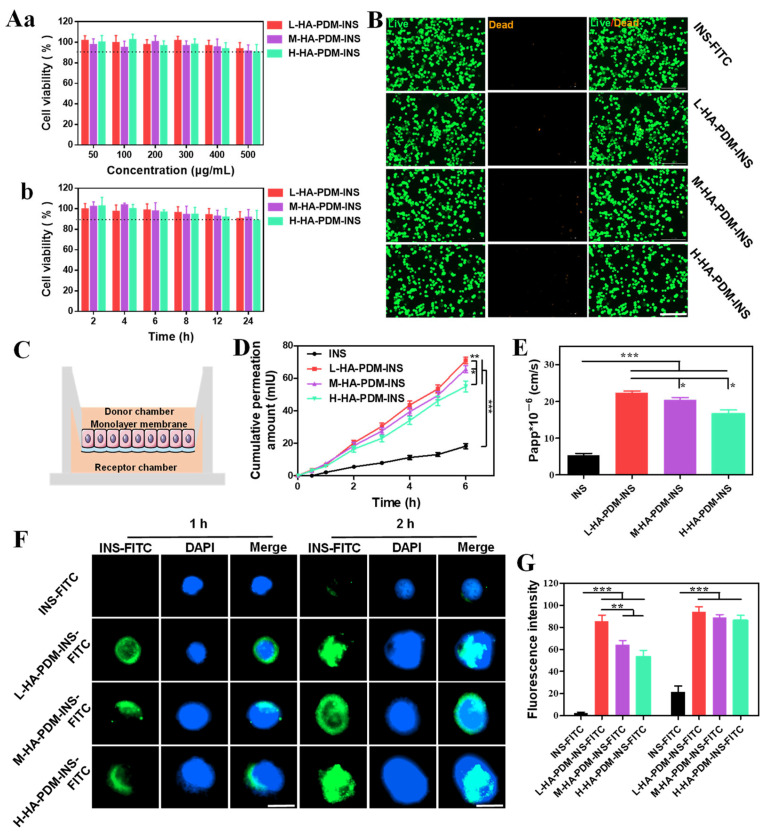
Effect of three nanoparticles with distinct concentrations (**A**(**a**)) and different time (**A**(**b**)) on the Caco-2 cells viability. (**B**) Live/dead staining of Caco-2 cells after incubation with the different nanoparticles for 1 d. The living cells were dyed green, and the dead cells were dyed orange, scale = 200 μm. (**C**) Schematic diagram of the Caco-2 cells monolayer barrier. (**D**) In vitro permeation studies of insulin from different nanoparticles across the Caco-2 cells monolayer barrier (X ± SD, n = 6, * *p* < 0.05, ** *p* < 0.01, *** *p* < 0.001). (**E**) Papp of insulin across Transwell after 6 h (X ± SD, n = 6, * *p* < 0.05, *** *p* < 0.001). (**F**) Cellular uptake of INS–FITC, L-HA–PDM–INS–FITC, M-HA–PDM–INS–FITC, and H-HA–PDM–INS–FITC in Caco-2 cells, scale = 20 μm. (**G**) Statistical analysis of the data in (**F**) (X ± SD, n = 6, ** *p* < 0.01, *** *p* < 0.001).

When Caco-2 cells were co-cultured with 500 μg/mL NPs for 24 h, the cell viability was still more than 80% (Figure 7A). The results of the live–dead cell staining also showed a large number of normally growing cells (green fluorescence) (Figure 7B), which indicates that a high concentration of NPs still had good biocompatibility when co-cultured with cells and did not affect the activity of cells.

#### 3.8.2. Transwell Experiments

TEER was directly related to the tightness of the cell junction. With the prolongation of culture time, the TEER value gradually increased (Figure S1), indicating that the tight junction barrier of cells was gradually increased. The integrity of the tight junction barrier can be visually reflected by measuring the TEER of Caco-2 cell monolayers to determine the transport pathway of INS. Figure 7C shows a schematic diagram of Caco-2 cell monolayers. There was no significant effect on TEER for 6 h in the free INS group, suggesting that INS was transported via the transcellular pathway, and Papp was only 5.35 ± 0.51 cm/s, which also suggests that free INS has poor transmembrane capacity. TEER values in the NPs group were consistently lower from 1–6 h, suggesting that tight junctions are involved in causing paracellular transport of INS. L-HA–PDM–INS, M-HA–PDM–INS, and H-HA–PDM–INS increased the Papp of INS to 22.26 ± 0.62, 20.38 ± 0.71, and 16.58 ± 1.11 cm/s, respectively, which increased by 4.16, 3.81, and 3.10 times, respectively. These results further demonstrated the prominent ability of NPs to promote INS transport, particularly L-HA–PDM–INS. The Papp of NPs decreased with increasing MW of HA, which may be due to the larger particle size and slow release rate of NPs prepared by high MW of HA.

#### 3.8.3. Cellular Uptake

The uptake results of Caco-2 cells after treatment with preparations containing the same INS–FITC concentration were showed in Figure 7F. Only weak green fluorescence was observed in the free INS group because INS is a biological macromolecule with large MW, large polarity, and strong hydrophilicity, and its passive diffusion and transmembrane ability are poor. In contrast, the encapsulation of INS–FITC into NPs results in a stronger intracellular green fluorescence as the NPs have a stronger negative charge and interact better with the cell membrane. The results of the uptake study were similar to those of transwell experiments. At 1 h, the uptake of L-HA–PDM–INS–FITC was more than that of the other two NPs. At 2 h, there was no significant difference in the uptake of the three NPs, probably because the uptake and translocation of the cells reached a balance. Figure 7G was a quantitative analysis of INS–FITC uptake by Caco-2 cells.

### 3.9. In Vivo Study

According to different administration methods, the blood glucose value of rats was detected to evaluate the activity of INS and its hypoglycemic effect in vivo.

#### 3.9.1. Pharmacodynamics

**Figure 8:**
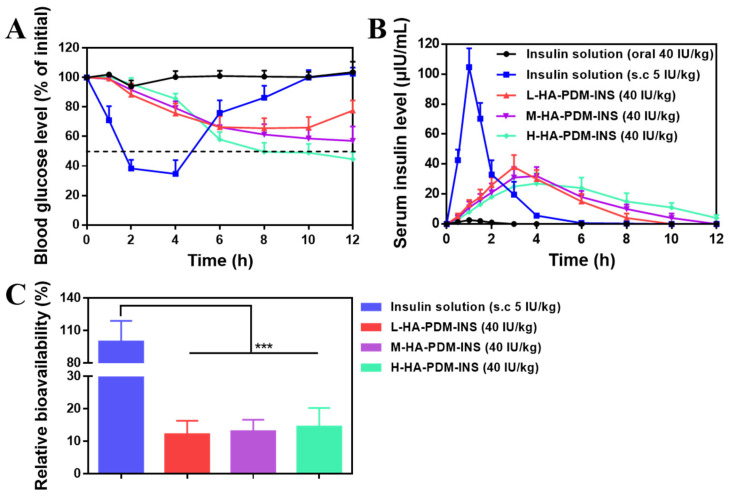
(**A**) Blood glucose level vs. time profiles of the diabetic rats following the administration of different formulations (X ± SD, n = 6). (**B**) Serum insulin level vs. time profiles of diabetic rats following the administration of different formulations (X ± SD, n = 6). (**C**) Relative bioavailability (X ± SD, n = 6, *** *p* < 0.001).

Figure 8A showed the blood glucose level changes in rats. Subcutaneous injection of INS solution could quickly take effect, and the blood glucose was reduced to the lowest level at 2–4 h, which was 34.67 ± 9.29% of the initial blood glucose, and then the blood glucose of diabetic rats gradually rose to the initial level. After oral administration of the INS solution, there was almost no significant change in the blood glucose level of the rats, which indicates that unprotected INS is easily inactivated by oral administration and cannot achieve a hypoglycemic effect [56]. Compared with injectable administration, the NPs of oral delivery had a better hypoglycemic effect in diabetic rats, with a slow hypoglycemic trend that did not cause hypoglycemia and kept their blood glucose levels at normal levels for 12 h. Among them, H-HA–PDM–INS had a long-term hypoglycemic effect. This also indicates that INS can maintain good biological activity in NPs. NPs act as carriers to protect and maintain INS stability in the hostile environment of the stomach and promote increased cellular permeability to INS, thereby improving cellular uptake and intracellular delivery. Although all three NPs showed good hypoglycemic effects at this dose, there were still two major problems. Firstly, the hypoglycemic effect of the NPs was slow. Secondly, the blood glucose level of the rats in the oral NPs group was still higher than the normal range. Therefore, the dosage of NPs should be further increased if the clinical level is to be achieved.

#### 3.9.2. Pharmacokinetic

The pharmacokinetic experiment was directed to study the absorption of NPs in vivo. The Insulin Assay Kit (built in Nanjing, China) was used to assay the INS concentration in serum at different time points and the pharmacokinetic curves are shown in Figure 8B. The pharmacokinetic parameters obtained from the drug–time curves are shown in Table 5. The results showed that the serum INS concentrations of rats in the INS solution group were consistently low. That is the oral uptake of free INS was quite low. The INS solution was absorbed rapidly after subcutaneous injection, reached its peak at 1 h, and then metabolized rapidly.

After oral administration of L-HA–PDM–INS, M-HA–PDM–INS, or H-HA–PDM–INS NPs, the relative bioavailability of INS increased to 12.27%, 13.16%, and 14.62%, respectively (Figure 8C). Blood glucose level—area on time curve (AAC) and Pharmacological activity (PA)—increased with increasing MW of HA, which is consistent with the results of the resistance to protease degradation assay. This phenomenon indicates that the NPs prepared with high MW of HA had a stronger protective capacity and a more significant sustained release effect, which prolonged the retention time of NPs at the absorption site and increased the oral absorption performance of INS.

### 3.10. Safety Evaluation

**Figure 9:**
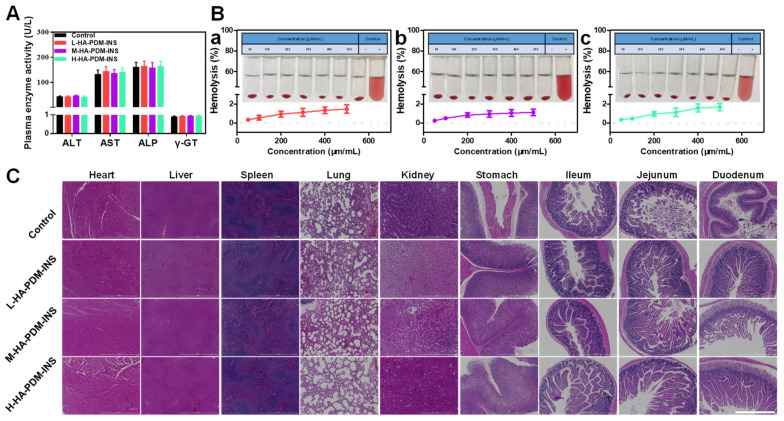
Safety. (**A**) Biomarkers of ALT, AST, ALP, and γ-GT from healthy rats after 7 d treatment with L-HA–PDM–INS, M-HA–PDM–INS, and H-HA–PDM–INS (X ± SD, n = 6). (**B**) Hemolysis assay with red blood cells exposed to L-HA–PDM–INS (**a**), M-HA–PDM–INS (**b**), and H-HA–PDM–INS (**c**) (X ± SD, n = 3). (**C**) H&E staining of the main organs (heart, liver, spleen, lung, kidney, and intestinal) harvested from different groups, scale = 1000 μm.

After 7 d of continuous oral administration at a high dose, the rats still exhibited normal appearance and behavior. As shown in Figure 9A, compared with the control group, there were no significant changes in the activities of ALT, AST, ALP, and γ-GT (*p* > 0.05). No significant hemolysis occurred at different concentrations of NPs, and the hemolysis rate was less than 1.8% (Figure 9B). According to the American Society for Testing and Materials, hemolysis of 2% or less is considered to be non-hemolysis for biomaterials. The H&E results showed normal morphology of the main organs (heart, liver, spleen, lung, kidney, and stomach) and no significant abnormalities in the villi of the duodenum, jejunum, and ileum (Figure 9C). There was no inflammation, mucosal erosion, foreign bodies, and other abnormal phenomena, which practically completely maintained the integrity and health of the intestine. Therefore, we believe these NPs are nontoxic and can be used for further study.

## 4. Discussion

For oral INS delivery, we developed INS-loaded pH-responsive and mucoadhesive NPs with different MW of HA and PDM. Depending on pH, HA–PDM–INS remained stable in the gastrointestinal environment. HA helps NPs adhere to the intestinal epithelium and may be a good choice to promote the interaction between the carrier and the intestinal wall, which prolongs the retention time of HA–PDM–INS at the absorption site and increases the dose across the intestinal mucosa. NPs are transported through the intestinal epithelium to reach systemic circulation and release bioactive INS.

In this study, the formulation and preparation of NPs were investigated using the single-factor method. Three HA–PDM–INSs with suitable particle sizes, stable Zeta potentials, and good roundness were obtained. DM is a water-soluble cationic monomer that can be polymerized to form PDM via KPS in an aqueous solution. HA is a polyanionic electrolyte that can assemble with cationic compounds to form complexes via electrostatic interaction.

Although HA has been widely used in drug delivery, only a few studies have focused on the effects of the MW on the properties of delivery systems. The MW of HA was found to have an influence on the formulation and preparation process of HA–PDM. As the MW of HA increases, the amount of initiator (KPS) required increases, and the reaction temperature increases, possibly because the viscosity of HA increases with increasing MW, and the initiator and reaction temperature can reduce the viscosity of the polymer. When the MW of HA increased, the DL of HA–PDM–INS also increased. This is due to the fact that during the self-assembly process, the high MW of HA occupies a larger space and carries more negative charges, which can adsorb many INS and improve the encapsulation performance.

The encapsulated of INS in NPs prevents contact between INS and the protease, which in turn resists the degradation of INS by the protease. There are two possible reasons for the ideal protection provided by HA–PDM–INS. (1) INS was prepared in a hydrochloric acid solution with a pH below the isoelectric point of INS. At this time, INS and PDM were positively charged. On the other hand, HA contains a large number of –OH and –COOH and has a negative charge under weakly acidic conditions [53]. INS and HA are attracted to each other via electrostatic interactions. The INS is thus closely bound to the carrier and does not leak easily. (2) At a low pH, the carboxyl groups of HA were protonated [57]. An increasing number of carboxyl groups form intermolecular hydrogen bonds, which can lead to tight contraction of the polymer chain segments and highly protected INS in the NPs, thus reducing INS leakage. When the MW of HA increased, the space occupied by HA increased, the negative charge increased, and the internal space became more compact. The NPs system was more stable and could maintain its INS structure for a long time.

The pH-responsive release of NPs was mainly due to the presence of PDM and HA. To understand the effect of pH on INS release, in vitro release experiments were performed at different pH (1.2, 6.8, and 7.4). At pH 1.2, the swelling rate of HA–PDM–INS was low. This was due to the ionization of PDM in acidic media, where the –N(CH_3_)_2_ group was fully protonated to –NH^+^–(CH_3_)_2_ [58]. –NH^+^–(CH_3_)_2_ forms intermolecular hydrogen bonds with the –OH and –COOH in HA, which contracted the network structure of the delivery system and limited the release of INS. The network structure became tighter as the MW of HA increased. At pH 6.8 and 7.4, the degree of protonation of the tertiary amine group decreased [59], and the number of intermolecular hydrogen bonds decreased, leading to an increase in the swelling of the HA–PDM–INS network. The highest release was observed at pH 7.4, which was due to the complete deprotonation of the –NH^+^–(CH_3_)_2_ group in alkaline media. The repulsion between –NH^+^–(CH_3_)_2_ and the electrostatic interactions was disrupted [17], causing the HA–PDM chain to expand. When the MW of HA was low, the expansion rate was higher, and the HA–PDM system was looser, thus INS was released to the maximum extent. Du et al. [60] also concluded that for HA matrix tablets, drug release results were consistent with swelling and erosion studies, whereas swelling and erosion rates were related to the MW of HA, ionic strength, and pH of the medium. HA has adhesive and penetration-promoting functions that prolong the retention time of NPs in the intestine, promote INS uptake by the epithelial cells, and increase the amount of INS entering the bloodstream.

The carboxyl or sulfate groups of mucin render it negatively charged. By encapsulating INS inside negatively charged hydrophilic HA, electrostatic and hydrophobic interactions between the NPs and mucins can be avoided. Mucosal fluidity can be enhanced through charge repulsion, which greatly weakens the adsorption of mucin on NPs and improves their permeability. As the MW of HA increased, more HA was bound to INS and PDM, thereby reducing the repulsion of NPs from mucin. Therefore, as the MW of HA increased, the permeation of the NPs decreased. The amount of permeation is not only related to the potential but also the size of the NPs. The permeability of the INS decreased with increasing particle size. In addition, NPs with hydrophilic properties are more likely to penetrate mucus [61].

The in vivo pharmacodynamic and pharmacokinetic properties of HA–PDM–INS were consistent with the in vitro results in promoting the oral absorption of INS. Bioactive INS binds to INS receptors on the cell surface, allowing glucose transporter proteins to transport glucose into the cells. In adipose tissue, INS prevents the breakdown of triglycerides into free fatty acids, which can be used as fuel, and facilitates the absorption of glucose for storage [62]. In type I diabetes, the body’s immune cells destroy insulin-producing pancreatic beta cells, preventing glucose from entering fat or muscle cells, which in turn boosts the production of adenosine triphosphate. In our study, the delivered INS maintained its biological activity after crossing the Caco-2 monolayer. This is encouraging, as one of the most challenging issues in oral INS delivery is preventing the degradation of INS during absorption, allowing it to remain biologically active. NPs can avoid the influence of an acidic environment in the stomach, protect INS over a wide pH range, release INS slowly in the intestine and blood, promote the uptake of INS by intestinal epithelial cells, improve the oral bioavailability of INS, and maintain a long-term hypoglycemic effect. Rapid clearance of the drug from the site of absorption is also considered a barrier to INS absorption [63]. Therefore, increasing the retention time of INS in the mucosa may lead to better bioavailability, and encapsulation of INS into mucoadhesive NPs may help improve bioavailability [27]. Similarly, it has been suggested that mucosal adhesion of the carrier is an important property for determining the bioavailability of poorly absorbed drugs [64]. We found that NPs prepared with a high MW of HA had a stronger protective capacity and a more significant sustained release effect, which prolonged the retention time of NPs at the absorption site and increased the oral absorption performance of INS.

Of course, the NPs we prepared also have some shortcomings, including large oral dose, low DL, and EE. This is a considerable point, since the EE of the developed NPs is crucial from a cost-effective point of view.

## 5. Conclusions

In this study, pH-responsive and mucoadhesive NPs were developed with different MW of HA and PDM for oral INS delivery. These HA–PDM NPs have prominent biocompatibility, protect the delivery of INS in the gastrointestinal tract, enhance the uptake of INS by cells, and improve oral bioavailability regardless of the MW of HA. Among them, L-HA–PDM had the high permeability, whereas M-HA–PDM had protection and delivery efficiency. These simple, low-cost, and environmentally friendly NPs are worthy of further development and industrial production. Next, we will study the effects of these NPs on INS delivery with other mammals.

## Figures and Tables

**Table 1:** Single-factor investigation.

	HA–PDM (L, M, and H)
DM (μL)	100
	300
	500
KPS (mL)	0.5
	1.5
	2.5
Stirring speed (rpm)	1040
	1300
	1560
Temperature (°C)	50
	60
	70

**Table 2:** Single-factor optimization: process and formulation.

		Value	Size	PDI	Zeta
L-HA–PDM	DM (μL)	100	445.99 ± 16.94	0.51 ± 0.16	−36.98 ± 2.41
		300	272.53 ± 8.32	0.32 ± 0.04	−31.64 ± 3.09
		500	276.06 ± 10.42	0.29 ± 0.04	−32.08 ± 1.98
	KPS (mL)	0.5	340.11 ± 13.16	0.46 ± 0.06	−37.29 ± 2.19
		1.5	255.68 ± 12.94	0.26 ± 0.05	−31.58 ± 1.97
		2.5	522.62 ± 14.39	0.33 ± 0.13	−23.42 ± 2.05
	Stirring speed (rpm)	1040	577.09 ± 15.92	0.34 ± 0.13	−32.27 ± 1.97
		1300	324.18 ± 11.68	0.22 ± 0.08	−31.64 ± 1.67
		1560	451.67 ± 9.97	0.21 ± 0.07	−34.92 ± 2.21
	Temperature (°C)	50	250.48 ±17.97	0.17 ± 0.07	−42.02 ± 4.02
		60	286.22 ± 18.66	0.15 ± 0.11	−40.68 ± 4.19
		70	445.99 ± 16.94	0.51 ± 0.16	−36.98 ± 2.41
M-HA-PDM	DM (μL)	100	534.02 ±25.64	0. 62 ± 0.18	−33.96 ± 2.89
		300	762.46 ± 19.64	0.54 ± 0.13	−36.39 ± 2.06
		500	453.04 ± 15.62	0.32 ± 0.09	−34.88 ± 2.14
	KPS (mL)	0.5	728.25 ± 17.69	0.33 ± 0.11	−36.92 ± 2.01
		1.5	443.11 ± 15.21	0.28 ± 0.09	−38.57 ± 1.97
		2.5	412.67 ± 12.71	0.23 ± 0.08	−35.04 ± 2.15
	Stirring speed (rpm)	1040	292.07 ± 11.29	0.24 ± 0.05	−33.84 ± 2.67
		1300	264.82 ± 11.06	0.16 ± 0.08	−34.02 ± 2.81
		1560	437.43 ± 10.11	0.21 ± 0.07	−38.59 ± 2.09
	Temperature (°C)	50	452.39 ± 13.42	0.19 ± 0.08	−27.99 ± 1.87
		60	262.59 ± 10.97	0.15 ± 0.04	−36.52 ± 3.14
		70	269.55 ± 10.67	0.16 ± 0.04	−26.98 ± 1.62
H-HA–PDM	DM (μL)	100	452.37 ± 19.64	0.67 ± 0.21	−28.64 ± 2.61
		300	304.62 ±16.35	0.33 ± 0.16	−32.05 ± 2.01
		500	287.88 ± 12.62	0.47 ± 0.11	−28.64 ± 1.99
	KPS (mL)	0.5	557.41 ± 16.98	0.38 ± 0.10	−38.65 ± 2.23
		1.5	400.21 ± 14.62	0.32 ± 0.08	−41.02 ± 1.97
		2.5	239.11 ± 10.98	0.24 ± 0.05	−37.88 ± 2.09
	Stirring speed (rpm)	1040	306.13 ± 15.09	0.22 ± 0.09	−30.94 ± 2.64
		1300	332.82 ± 12.01	0.18 ± 0.05	−26.48 ± 1.96
		1560	277.98 ± 10.93	0.14 ± 0.04	−29.54 ± 1.89
	Temperature (°C)	50	592.59 ± 18.19	0.16 ± 0.07	−34.62 ± 3.01
		60	395.92 ± 10.67	0.15 ± 0.08	−31.82 ± 1.67
		70	275.24 ± 8.00	0.14 ± 0.05	−25.61 ± 1.98

**Table 3:** Optimum process and formulation results.

	DM (μL)	KPS (mL)	Stirring Speed (rpm)	Temperature (°C)
L-HA–PDM	300	1.5	1300	50
M-HA–PDM	500	2.5	1300	60
H-HA–PDM	300	2.5	1560	70

**Table 4:** Mathematical models of the regression for in vitro release profiles of preparations.

		Zero Order	First Order	Higuchi	Ritger–Pappas	
Mathematical Equation		Q_t_ = k_0_t + Q_0_	Log Q_t_ = LogQ_0_ − k_0_t/2.303	Q_t_ = kHt^1/2^	M_t_/M_∞_ = kt^n^	
		R^2^	R^2^	R^2^	R^2^	n
pH 1.2	L-HA–PDM–INS	0.8336	0.9983	0.9766	0.9766	0.4945
	M-HA–PDM–INS	0.8660	0.9983	0.9822	0.9823	0.5314
	H-HA–PDM–INS	0.8411	0.9968	0.9772	0.9768	0.5049
pH 6.8	L-HA–PDM–INS	0.8164	0.9890	0.9500	0.9492	0.5219
	M-HA–PDM–INS	0.9074	0.9781	0.9303	0.9540	0.7017
	H-HA–PDM–INS	0.9468	0.9843	0.9261	0.9709	0.7783
pH 7.4	L-HA–PDM–INS	0.8320	0.9950	0.9636	0.9630	0.5199
	M-HA–PDM–INS	0.9243	0.9959	0.9672	0.9825	0.6504
	H-HA–PDM–INS	0.9351	0.9942	0.9593	0.9818	0.6864

**Table 5:** Pharmacodynamic parameters after administrating different insulin samples in rats. (X ± SD, n = 6).

	S.C	Oral	L-HA–PDM–INS	M-HA–PDM–INS	H-HA–PDM–INS
Dose (IU/kg)	5	40	40	40	40
AUC_0–∞_ (μIU/mL·h)	180.31 ± 32.31		177.07 ± 83.24	189.83 ± 72.16	210.90 ± 113.06
MRT_0–∞_ (h)	1.63 ± 0.07	-	3.96 ± 0.50	4.88 ± 0.82	6.17 ± 0.39
VRT_0–∞_ (h^2^)	2.13 ± 0.01	-	7.22 ± 5.89	10.82 ± 5.61	21.88 ± 9.12
t_1/2z_ (h)	1.27 ± 0.09	-	1.88 ± 1.06	2.36 ± 0.33	3.35 ± 1.11
T_max_ (h)	1	-	3	4	4
CL_z/F_ (L/h/kg)	28.39 ± 4.30	-	183.89 ± 91.37	166.22 ± 64.64	143.90 ± 79.58
C_max_ (μIU/mL)	104.67 ± 10.61	-	38.01 ± 11.31	31.98 ± 8.49	27.03 ± 9.89
AAC	330.1	17.3	293.2	324.8	377.1
PA (%)	100	0.66	11.10	12.30	14.28

AUC: area under concentration—time curve; MRT: mean residence time; VRT: variance of residence time; t_1/2z_: elimination half-life; T_max_: time to reach maximum concentration; CL_z/F_: clearance rate; C _max_: maximum concentration; AAC: blood glucose level—area on time curve, (area above the curve); PA: pharmacological activity.

## Data Availability

The data presented in this study are available in this article (and Supplementary Materials).

## Supplementary Materials

The following supporting information can be downloaded at: https://www.mdpi.com/article/10.3390/pharmaceutics15030820/s1, Figure S1: Calibration curve of INS. Figure S2: Cell TEER curve during cultivation. Table S1: Detection method of INS. Table S2: Freeze-drying protocol of the frozen NPs. Reference [37] are cited in the supplementary materials.

## Abbreviations

HA: hyaluronic acid; DM: 2-(Dimethylamino)ethyl methacrylate; PDM: Poly((2-dimethylamino)ethyl methacrylate); INS: insulin; NPs: nanoparticles; MW: molecular weight; KPS: potassium persulfate; PDI: polydispersity indexes; EE: encapsulation efficiency; DL: drug loading; TEM: transmission electron microscopy; SGF: simulated gastric fluid; SIF: simulated intestinal fluid; CLSM: confocal laser scanning microscope; TEER: transepithelial electrical resistance; DAPI: 4′,6-diamidino-2-phenylindole.
