# Supplementary material for: pH-Responsive and Mucoadhesive Nanoparticles for Enhanced Oral Insulin Delivery: The Effect of Hyaluronic Acid with Different Molecular Weights
Source: Pharmaceutics. 2023 Mar 2;15(3):820. doi: 10.3390/pharmaceutics15030820 (PMC10056758; doi:10.3390/pharmaceutics15030820)
Supplement: Supplementary file 1 [file pharmaceutics-15-00820-s001.zip › pharmaceutics-2170846-supplementary.pdf]

Supplementary Materials

pH-Responsive and Mucoadhesive Nanoparticles for Enhanced Oral Insulin Delivery: The Effect of Hyaluronic Acid with Different Molecular Weights

Shuangqing Wang [1,2,†], Saige Meng [3,†], Xinlei Zhou [1], Zhonggao Gao [1,2,*] and Ming Guan Piao [1,*]

1 Key Laboratory of Natural Medicines of the Changbai Mountain, Ministry of Education, College of Pharmacy, Yanbian University, Yanji 133002, China
2 State Key Laboratory of Bioactive Substance and Function of Natural Medicines, Department of Pharmaceutics, Institute of Materia Medica, Chinese Academy of Medical Sciences and Peking Union Medical College, Beijing 100050, China
3 Department of Pharmacy, No. 73 Group Military Hospital of PLA, Xiamen 361003, China
* Correspondence: zggao@imm.ac.cn (Z.G.); mgpiao@ybu.edu.cn (M.G.P.)
† These authors contribute equally to this work.

Analytical method of INS

The concentration of INS was determined by Agilent HPLC, which consisting of 1290 Flexible Pump, 1290 Multisampler and 1290 VWD, detection method, as shown in Table S1. Figure S1 is the calibration curve of INS. The methods were validated in terms of specificity, linearity, accuracy, precision, detection limit, and quantification limit.

Table S1 Detection method of INS

Chromatographic column	Mobile phase	Velocity of flow	Sample volume	Column temperature	Wavelength
Thermo Hypersil BDS C18 (150×4.6 mm, 5 μL)	Anhydrous sodium sulfate: acetonitrile (72: 28)	1 mL/min	20 μL	40℃	198 nm

Freeze-drying process

Briefly, after the INS-loaded NPs were prepared, the reaction solution was centrifuged at high speed (18,000 rpm, 30 min, 4 °C). At this time, the reaction solution contained INS-loaded NPs, unloaded INS (free INS) and unreacted monomers. At the end of high speed centrifugation, the upper layer of the solution contains unloaded INS and unreacted monomers. 2.5% (W/V) trehalose was used as the cryoprotectant in order to prevent aggregation [1]. NPs were mixed with 2.5% trehalose and then frozen in a refrigerator at -40 ℃ overnight. Finally, the samples were lyophilized by using an EPSILON1-4LSC freeze dryer (Martin Christ, Germany) to acquire freeze-dried NPs. Pressure (MPa) was 0.01 MPa. The freeze-drying protocol was shown in Table S2.

Table S2 Freeze-drying protocol of the frozen NPs.

Temperature (°C)	**-45**	**-25**	**-25**	**-10**	**-10**	**0**	**25**
Time (h)	0	1	6	7	11	17	30

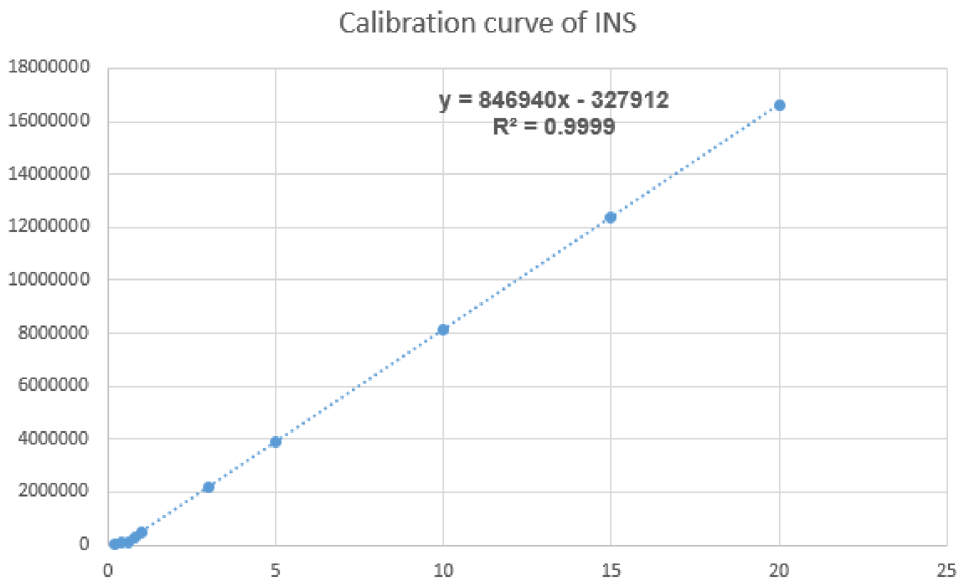

Figure S1 Calibration curve of INS

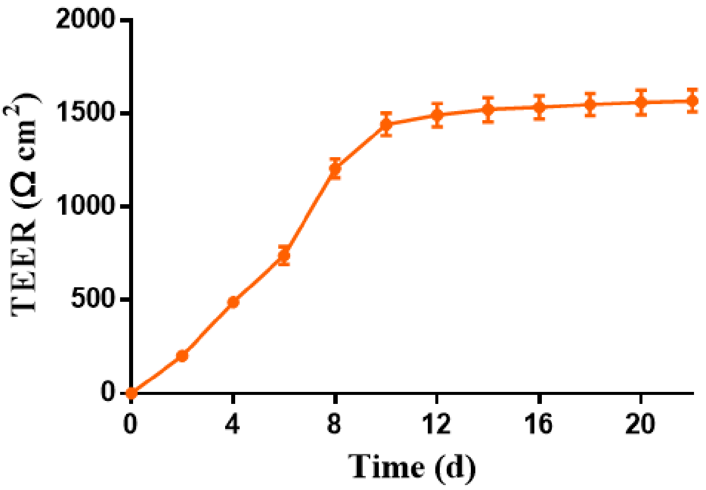

Figure S2 Cell TEER curve during cultivation

1. Fan, W.; Xia, D.; Zhu, Q.; Li, X.; He, S.; Zhu, C.; Guo, S.; Hovgaard, L.; Yang, M.; Gan, Y. Functional Nanoparticles Exploit the Bile Acid Pathway to Overcome Multiple Barriers of the Intestinal Epithelium for Oral Insulin Delivery. *Biomaterials* **2018**, *151*, 13–23, doi:10.1016/j.biomaterials.2017.10.022.
